# Animal Fat as a Substrate for Production of n-6 Fatty Acids by Fungal Solid-State Fermentation

**DOI:** 10.3390/microorganisms9010170

**Published:** 2021-01-14

**Authors:** Ondrej Slaný, Tatiana Klempová, Volha Shapaval, Boris Zimmermann, Achim Kohler, Milan Čertík

**Affiliations:** 1 Faculty of Chemical and Food Technology, Institute of Biotechnology, Slovak University of Technology, Radlinského 9, 812 37 Bratislava, Slovakia; tatiana.klempova@stuba.sk (T.K.); milan.certik@stuba.sk (M.Č.); 2 Faculty of Science and Technology, Norwegian University of Life Sciences, Postbox 5003, 1432 Ås, Norway; volha.shapaval@nmbu.no (V.S.); boris.zimmermann@nmbu.no (B.Z.); achim.kohler@nmbu.no (A.K.)

**Keywords:** solid-state fermentation, *Umbelopsis isabellina*, γ-linolenic acid, β-carotene, Fourier transform infrared (FTIR) spectroscopy, animal fat by-product

## Abstract

The method of solid-state fermentation (SSF) represents a powerful technology for the fortification of animal-based by-products. Oleaginous Zygomycetes fungi are efficient microbial cell factories used in SSF to valorize a wide range of waste and rest cereal materials. The application of this fermentation technique for utilization and biotransformation of animal-based materials represents a distinguished step in their treatment. In this study, for the first time, the strain *Umbelopsis isabellina* CCF2412 was used for the bioconversion of animal fat by-products to the fermented bioproducts enriched with n-6 polyunsaturated fatty acids, mainly γ-linolenic acid (GLA). Bioconversion of both cereals and the animal fat by-product resulted in the production of fermented bioproducts enriched with not just GLA (maximal yield was 6.4 mg GLA/g of fermented bioproduct), but also with high yields of glucosamine. Moreover, the fermentation on the cornmeal matrix led to obtaining bioproduct enriched with β-carotene. An increased amount of β-carotene content improved the antioxidant stability of obtained fermented bioproducts. Furthermore, the application of Fourier-transform infrared spectroscopy for rapid analysis and characterization of the biochemical profile of obtained SSF bioproducts was also studied.

## 1. Introduction

The increasing worldwide demand for meat products is accompanied by the emergence of new challenges. Meat production is linked to generating large amounts of animal fat by-products (millions of tons) and the majority of challenges are associated with inevitable post-production storage or fully ecological liquidation of accumulated by-products [1,2]. Animal fat by-products or waste fractions may cause devastating physical effects on the ecosystem, produce rancid odors, or form toxic products that can linger in the environment for many years. The result of these difficulties is gradually a disadvantage of the entire process due to the deterioration of the economic balance and sustainability of the whole meat production process. Nowadays, searching for alternative ways for solving such problems with cheap, affordable, and easy to perform processes of degradation of animal fat by-products or their conversion into highly valuable and environmentally acceptable compounds is one of the most emerging technological questions. Different bioprocesses present novel ways to solve such problems more effectively.

At present, several studies describe the possibility of biodiesel production from animal fat by-products as input material with relatively high efficiency [2,3,4]. However, this fat-based by-product may not be just a suitable matrix for its direct transesterification to biodiesel. Using animal fat as a substrate for different fermentation processes may lead to the production of a wide spectrum of different high-value bioproducts. Meanwhile, utilization and biotransformation of various exogenous oils (i.e. linseed, sunflower, palm, olive, or peanut oil) that served as precursors for biosynthesis of a wide variety of secondary fungal-specific metabolites have been described previously [5,6,7,8,9,10]. A specific fermentation process called solid-state fermentation (SSF) is a well-described process using a solid matrix and almost no water, accompanied by a high-production capacity [11,12]. Application of SSF with Zygomycetes fungi as a main production cell factories allow acquiring of bioproducts enriched with various long-chain unsaturated fatty acids [6,13,14] and also other specific valuable products, such as glucosamine [15], carotenoid pigments [6,16,17,18], coenzymes [19], etc. Furthermore, the process of SSF often leads to the increased enzyme activity (e.g. amylase or lipase) in obtained fermented products [20,21,22]. Possible application of animal fat by-products as an input substrate for SSF is therefore advantageous not only in terms of its biodegradation but also in possible biotransformation to other high value-added products.

As already mentioned, the application of various fungal or yeast strains for exogenous oil or fat utilization has been reported. After all, mainly yeasts of genus *Yarrowia* are used for effective biotransformation of various animal-based fat-substrates (i.e. cod liver and beef tallow [23], pork lard [24], mutton fat [25], goat tallow [26], or animal-based stearin [27,28]). However, all of these biotransformation processes are performed under the condition of the submerged fermentation. Our team pioneered and described the process of SSF using solid animal fat by-product substrates [13]. Using Zygomycetes strain *Mortierella alpina* CCF2861, bioproducts with the increased level of ω-6 fatty acids was obtained. Moreover, the tested fungal strain was able to some extent biodegrade animal fat material and transform it into valuable fungal specific bioproduct. Hence, the logical next step is to investigate a possible application of other filamentous fungi for the SSF process of animal fat materials. One of the key goals of this study is to evaluate previous findings, improve product yields of desired metabolites, and extend the process of SSF to produce other valuable compounds.

Fungus *Umbelopsis isabellina*, used in this study, is a relatively well-known Zygomycetes species for application in bioremediation [29,30] and fermentation processes [16,17,31]. Its ability to effectively produce long-chain fatty acid and carotenoid pigments was described previously [16,31,32]. However, an important characteristic of *U. isabellina* is mostly its ability to grow in highly specific conditions during SSF. This includes in particular lower water activity or impaired oxygen transfer [11,33]. At the same time, *U. isabellina* growing under these conditions also form specific microbial lipophilic metabolites mentioned above [34]. Effective production of such high value-added fermented products with no need for their fractionation predetermines it for possible direct industry usage, e.g. in the feed or food industry [17,35].

The presented study is the first one investigating the possibility to develop effective bioconversion of animal fat by-products into lipid-rich fermented bioproducts by SSF using *U. isabellina*. Furthermore, the study also describes the increased antioxidant activity of these bioproducts. Moreover, a novel approach of analysis of SSF bioproducts using Fourier transform infrared (FTIR) spectroscopy reported recently [13] as an alternative method for the accurate monitoring of SSF processes was also confirmed.

## 2. Materials and Methods

### 2.1. Production Microorganism

The oleaginous fungus *Umbelopsis isabellina* CCF2412 obtained from the Culture Collection of Fungi (CCF, Charles University, Prague, Czech Republic) was kept on potato-dextrose agar media (Carl Roth, Karlsruhe, Germany) at 4 °C and regularly re-inoculated every 3 months.

### 2.2. Inoculation of Solid-State Fermentation Substrate and Fermentation Conditions

The spore suspension for the inoculation of the fermentation substrate was prepared from 7-days old culture grown on a PDA medium. Spores were washed using sterile aqueous solution of 0.05% Tween^®^ 40 (Sigma-Aldrich, St. Louis, MO, USA). The obtained spore suspension was diluted to achieve a final concentration of 10^6^ spores/mL.

Static SSF cultivations were performed in the microporous high-density polyethylene bags (20 × 30 cm) containing 20 g of the dry cereal-based matrix to which various amounts of animal fat material (Norilia, AS, Oslo, Norway) mixed with Tween^®^ 40 was added. SSF experiments have been performed using wheat bran (WB; Mill Pohronský Ruskov, Slovakia) and cornmeal (CM; Amylum Slovakia, Boleráz, Slovakia) as a SSF matrix. Until further processing, both cereal substrates were stored at 25 °C in an opaque sealed container.

The chemical composition of animal fat (AF) material (Norilia AS, Oslo, Norway) used is summarized in Table 1.

Both cereal substrates were processed according to the method described by Slaný et al. [13]. The composition of cereal substrates mixtures with Tween^®^ 40 and animal fat is summarized in Table 2. Obtained sterilized substrate mixtures (including 20 g of cereal substrate and the relevant quantity of distilled water or Tween^®^ 40 and animal fat material emulsion according to Table 2) were inoculated with 4 mL of spore suspension of *U. isabellina*. The static solid-state fermentation was run for 7 days at 28 °C. All experiments were performed in three independent biological replicates.

### 2.3. Preparation of AF Emulsion

Animal fat emulsions were prepared by mixing distilled water, Tween^®^ 40, and animal fat material using a combination of heating, stirring, and sonication by VWR USC300T ultrasonic homogenizer (VWR International, Radnor, PA, USA) according to Slaný et al. [13].

### 2.4. Humidity and Substrate Utilization Analysis

The humidity of the collected fermented bioproducts was measured by Moisture Analyzer Radwag 50/1. R. Afterwards, the fermented bioproducts were dried at 65 °C until a constant weight was achieved, approx. after 24 h. The wheat bran substrate utilization was calculated from the weight difference of dry non-fermented substrate (control) and dry fermented bioproducts. All bioproduct samples were homogenized using a blender and stored at 25 °C in sterile Falcon tubes.

### 2.5. Estimation of Fungal Biomass and Glucosamine Amount in the Fermented Bioproduct

To estimate the amount of fungal biomass in the fermented bioproducts, the method based on the estimation of glucosamine (GlcN) content was used. At first, alkali insoluble material (AIM) was prepared from obtained fermented bioproducts, according to a modified method based on the assay described by Zamani et al. [36]: NaOH solution (0.5M, 3 mL) was added to the samples of fermented bioproduct (100 mg) and the mixtures were heated overnight at 90 °C. Afterward, samples were centrifuged (5000 rpm, 10 min), and washed 5 times with distilled water (10 mL). Supernatants were removed and obtained AIMs were dried (36 h; 75 °C) until the constant weight of samples was achieved. Further, AIM samples were hydrolyzed by HCl solution and treated according to the method described by Slaný et al. [13]. Glucosamine (GlcN) content which correlates with the fungal biomass (FBM) amount was calculated according by using a standard calibration curve. Each analysis was performed in three independent technical replicates.

### 2.6. Complex Lipid and Carotenoid Pigments Extraction

Lipid/pigments extracts were obtained by the modified method described by Folch et al. [37]. Then, 1 g of dry fermented bioproduct was thoroughly homogenized by the mortar. 2-step extraction using chloroform/methanol solution (2:1 (v/v)) was performed in a total duration of 1.5 h with occasional stirring. Afterward, the mixtures were filtered, and 1.2-fold of the total extract volume of distilled water was added. Obtained mixtures were stirred for 1 minute and centrifuged (3500 rpm, 5 min) to obtain full phase separation. The chloroform layer containing lipids and pigments was dried using anhydrous Na_2_SO_4_ and evaporated under a vacuum. Obtained lipid/pigments extract was suspended in the 1 mL mixture of hexane/chloroform (9:1).

### 2.7. Analysis of Carotenoid Pigments by High-Performance Liquid Chromatography (HPLC)

Carotenoid pigments were analyzed by HPLC according to Klempová et al. [16]. The carotenoid pigments were identified and quantified by using authentic standards (Sigma-Aldrich, USA) and analyzed by ChemStation B 01 03 (Agilent Technologies, Santa Clara, CA, USA). Quantification of β-carotene was calculated from the standard calibration curve of pure β-carotene standard (Sigma-Aldrich, Karlsruhe, Germany).

### 2.8. Analysis of Fatty Acid Profile and Content in Fermented Bioproduct

Fatty acid (FA) from obtained fermented bioproducts were converted into their methyl esters (FAMEs) by the modified method of Čertík and Shimizu [38], according to the method described by Slaný et al. [13]. Obtained FAMEs were subsequently analyzed by gas chromatography according to the method described by Gajdoš et al. [39]. Identification of the individual FAMEs peaks was performed by comparison with authentic standards of C4–C24 FAME mixtures (Sigma-Aldrich, Karlsruhe, Germany). Quantitative evaluation of individual and total fatty acids was performed using an internal standard of heptadecanoic acid (C17:0, Supelco, Bellefonte, PA, USA) and calculated by ChemStation B 01 03 (Agilent Technologies, Santa Clara, CA, USA). Each analysis was performed in three independent technical replicates.

### 2.9. Analysis of Lipid Classes by Thin-Layer Chromatography (TLC)

Obtained total lipid extracts were loaded onto TLC silica plates 60 (Merck, Darmstadt, Germany) using CAMAG TLC Sampler 4 (CAMAG, Muttenz, Switzerland). TLC analysis was performed according to the method described by Gajdoš et al. [39]. Separated lipid fractions were analyzed densitometrically using CAMAG TLC Scanner 4 (CAMAG, Switzerland) and quantified using WinCATS software (CAMAG, Muttenz, Switzerland). Each analysis was performed in three independent technical replicates.

### 2.10. Antioxidant Activity Assay

Dried SSF bioproducts (0.5 g) were mixed with ethanol/distilled water (10 mL), stirred for 10 min, and subsequently centrifuged (5000 rpm, 5 min). Obtained supernatant (50 μL) was transferred into the cuvette and mixed with 2 mL of standard ABTS^+^ solution, which was prepared as follows: K_2_S_2_O_8_ (3.3 mg) was diluted in distilled water (5 mL). Afterward, ABTS standard (17.2 mg; Sigma Aldrich, St. Louis, MO, USA) was added to this solution, and the obtained mixture was incubated for 12 h at 25 °C. This solution was diluted (1:60 (v/v)) before use in the following reaction.

Estimation of ABTS^+^ concentration was performed by measuring the absorbance changes for 10 min at 730 nm. Final antioxidant activity is expressed as Trolox (Sigma Aldrich, St. Louis, MO, USA) equivalent in mmol/g of dried SSF bioproduct according to a standard calibration curve.

### 2.11. ATR-FTIR Spectroscopy Analysis

Fourier Transform Infrared (FTIR) spectroscopy was performed according to the method described by Slaný et al. [13] using a Vertex 70 FTIR spectrometer (Bruker Optik GmbH, Ettlingen, Germany) with a High Temperature Golden Gate ATR Mk II single-reflection attenuated total reflectance (ATR) accessory (Specac, Orpington, UK) to obtain a complex biochemical composition of the analyzed SSF bioproducts. All ATR-FTIR measurements were performed in five independent technical replicates, resulting in 180 spectra in total. Spectra were recorded in a region between and 600 and 7000 cm^−1^ with a resolution of 4 cm^−1^. Each spectrum was recorded as the ratio of the sample spectrum to the spectrum of the empty ATR plate. Recording of spectra was performed using the OPUS 7.5 software (Bruker Optic, Ettlingen, Germany).

### 2.12. Data Analysis

The fatty acid GC-FID, carotenoid pigments HPLC, antioxidant activity ABTS^+^, and GlcN data were analyzed by ANOVA using Microsoft Excel (Microsoft Office 365 software pack) equipped with a standard data analysis tool. Post-hoc testing was performed for the obtained ANOVA results using Tukey’s HSD test in programming language R and Python v. 3.7 using StatsModels libraries.

All FTIR-ATR spectra used were analyzed according to our previous study [13]. Region of 4000–600 cm^−1^ was selected as the spectral region containing all important bands distinctive for lipids, proteins, and polysaccharides. The pre-processing of FTIR-ATR data was performed by transforming to second-derivative spectra using the Savitzky−Golay algorithm with a polynomial of degree 2 and a window size of 11. The second-derivative spectra were pre-processed by extended multiplicative signal correction (EMSC). Subsequent PCA was performed for three independent spectral regions, lipid (3050–2800 cm^−1^ combined with 1800–1700 cm^−1^), protein (1700–1500 cm^−1^), and polysaccharide (1200–700 cm^−1^), using 11 principal components.

## 3. Results

The main aim of this study was to expand previous knowledge of our recently presented research on solid-state fermentation of animal fat by-products [13]. In the mentioned study, fungus *M. alpina* was described as an effective microbial cell factory for bioconversion of animal fat by-product materials. However, the success of such transformation was lowered with increasing animal fat by-product material concentration in the SSF substrates mixtures. Possible usage of different fungal strains, *Umbelopsis isabellina* CCF2412, for more effective and rapid utilization of used animal fat by-product material has been investigated in this study. The production ability of the tested strain was studied for GLA and β-carotene accumulation along with the estimation of GlcN amount and antioxidant activity of the obtained SSF bioproducts.

### 3.1. Wheat Bran Utilization and U.isabellina Growth Characteristics During SSF

Two types of SSF matrixes, wheat bran (WB)/cornmeal (CM), were mixed with various concentrations of Tween^®^ 40 and animal fat material to obtain SSF substrates (Table 1). Since emulsifier Tween^®^ 40 may impact the fungal growth and lipid accumulation metabolism, the effect of Tween^®^ 40 itself was investigated. Nonetheless, the presence of Tween^®^ 40 in the SSF system had no significant impact on the course of the fermentation process and fungal metabolism. Statistical analysis of all obtained data showed that regardless of its concentration in the SSF substrates, all observed growth parameters were in general fully comparable with the control substrate where no Tween^®^ 40 was added (*p*-value > 0.5; *F* parameter > *F*_crit_).

WB utilization with neither addition of Tween^®^ 40 nor animal fat was 17.3% (Table 3). However, an improved WB utilization ratio was observed with increasing concentration of Tween^®^ 40 in SSF substrate. The maximum utilization of WB of 21.8% was recorded in the case of 3% Tween^®^ 40 addition. On the contrary, the presence of animal fat in the SSF substrate has led to impaired availability of the cereal matrix which resulted in the slowdown of WB utilization. The worst WB matrix utilization of 8.9% was observed in the case of 30% addition of animal fat (Table 3). In the case of CM-based SSF, similar matrix utilization characteristics were observed. After animal fat addition, the utilization of CM decreased from approx. 25.0% (0% of animal fat addition) down to 6.6% (30% of animal fat addition) (Table 3).

Different utilization rates also resulted in changes in the percentage of humidity, pH value, and fungal growth characteristics (Table 3). In case of wheat bran-based SSF, the concentration of Tween^®^ 40 did not affected the humidity (values varied from 48.1–45.9%) neither FBM amount (values varied from 229.8–266.9 mg of FBM/g of bioproduct) with statistical importance (*p*-value > 0.5; *F* parameter > *F*_crit_). Moreover, increasing concentration of animal fat in the SSF system also resulted in lowering pH values from 5.3 (WB + 1% Tween^®^ 40) down to 4.5 (WB + 3% Tween^®^ 40 + 30% animal fat). The yield of FBM was also strongly affected by the concentration of animal fat in the SSF system. Increased animal fat addition to WB matrix resulted in the slowing of the fungal growth. FBM yields decreased from 229.8–266.9 mg FBM/g of fermented bioproduct in case of the WB-based substrates only with Tween^®^ 40 (0% of animal fat addition) down to 113.3 mg FBM/g of fermented bioproduct (WB + 3% Tween^®^ 40 + 30% animal fat) (Table 3).

Similarly, as cereals matrixes utilization characteristics, the percentage of humidity, pH value and FBM amount was also showed the same patterns in WB-based SSF and CM-based SSFs. The most significant differences were observed for FBM amounts. Along with the increased concentration of animal fat, the amount of FBM formed during the process of SSF decreased from 241.1 mg FBM/g of fermented bioproduct (0% of animal fat addition) down to 92.96 mg FBM/g of fermented bioproduct (30% of animal fat addition). Interestingly, the moderate addition (5%) of animal fat into CM matrix did not cause significant deceleration of fungal growth. In this case, the amount of FBM was 206.2 mg FBM/g of fermented bioproduct (Table 3).

The concentration of animal fat in the fermentation system also affected the water activity (a_w_) values. In general, the WB-based fermentation process decreased the a_w_ value. Moreover, with increasing concentration of animal fat, the a_w_ value was significantly lower for both fermented and non-fermented samples. However, in the case of CM-based fermentations, increased animal fat concentration led to rising a_w_ values (Table 3).

### 3.2. Fungal Lipids Accumulation and Lipid Profile

Since the strain *U. isabellina* CCF2412 was able to utilize available cereal matrixes with various effectiveness, that affected growth characteristics and fungal metabolism, different microbial lipid accumulation was also observed. Similar to previous data dealing with FBM growth or the humidity value, the percentage of lipid was not affected by the concentration of Tween^®^ 40 in the SSF substrate. Nevertheless, as it was expected, with increasing concentration of animal fat the percentage of total lipid significantly grew. The maximum percentage of total fatty acids (TFA; 23.4%) was observed in the non-fermented mixture of WB with 3% Tween^®^ 40 and 30% animal fat addition (Table 4). After the process of SSF, the concentration of total lipid slightly decreased down to 20.0%. This characteristic accompanied the WB-based fermentation process from the 0% of animal fat addition up to the highest animal fat concentration. On the other hand, the CM-based fermentation process led to increased TFA accumulation, regardless the concentration of animal fat added (Table 4).

TLC analysis of individual lipid classes proved that the value of animal fat addition to SSF substrates strongly affected the total lipid profile. A higher concentration of animal fat resulted in increased amounts of triacylglycerols and free fatty acids. On the contrary, polar lipid, 1,2-diacylglycerols, and esterified sterols content slightly decreased with an increased animal fat concentration in the SSF system (Table 5).

### 3.3. γ-Linolenic Acid Yield

**Figure 1:**
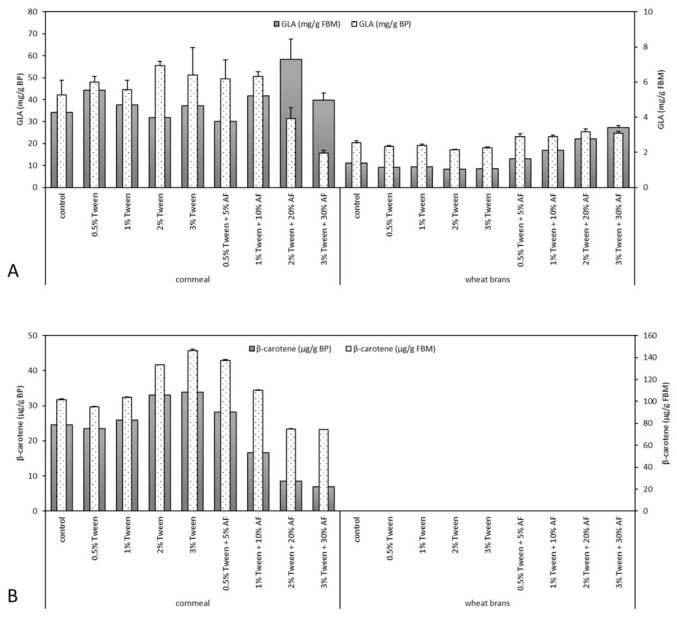
Content of γ-linolenic acid (GLA) (**A**) in fermented bioproducts (BP) and fungal biomass (FBM) and β-carotene yields (**B**) in BP and FBM. AF—animal fat by-product. Error bars showed a standard deviation, α = 0.05.

All SSF processes performed have proved the good ability of *U. isabellina* CCF2412 to form γ-linolenic acid (GLA, Figure 1). After the process of SSF, the amount of GLA reached the maximum yield of 3.2 mg GLA/g of fermented WB-based bioproduct (22.2 mg GLA/g of FBM) in the case of WB-based SSF with 20% of animal fat addition. The maximum yield of GLA in FBM after WB-based fermentation was reached even in the case of 30% animal fat addition (27.3 mg of GLA/g of FBM, Figure 1). For CM-based fermentations, the obtained yields of GLA were even higher. The maximal yield was detected in bioproducts obtained by fermentation with 10% of animal fat addition (6.4 mg GLA/g of fermented bioproduct) and in pure FBM by fermentation with 20% of animal fat addition (58.4 mg GLA/g of FBM, Figure 1).

### 3.4. β-Carotene Accumulation

*U. isabellina* CCF2412 is previously described as an effective producer of carotenoid pigments during both submerged and SSF processes [16,17]. However, during this study, only trace yields of β-carotene were recorded in the case of WB-based fermentation processes (Figure 1). On the other hand, in the case of CM-based fermentation processes, decent yields of β-carotene were reached. Nevertheless, the highest yield was reached in the case of 0% animal fat addition (33.9 μg/g of fermented bioproduct; 146.1 μg/g of FBM, Figure 1). Increasing concentration of animal fat resulted in decreasing accumulation of β-carotene in fermented bioproducts.

### 3.5. Glucosamine Accumulation

**Figure 2:**
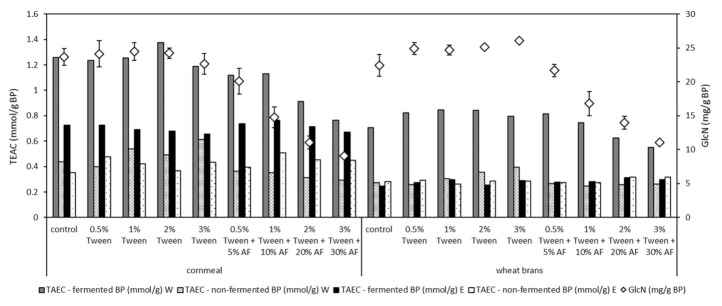
Yields of glucosamine (GlcN) in mg/g of fermented bioproducts and antioxidant activity (TEAC) expressed as Trolox equivalent in mmol/g of dried SSF bioproduct. W—water leachates, E—ethanol leachates, AF—animal fat by-product. Error bars showed a standard deviation, α = 0.05.

Glucosamine represents another high-value fungal product. *U. isabellina* CCF2412 was able to reach high yields of this specific metabolite. Glucosamine yields varied from 11.0 to 26.0 mg of glucosamine/g of WB-based fermented bioproduct (Figure 2). The highest value of glucosamine amount was reached in the case of WB mixture with 3% of Tween^®^ 40 and 0% of animal fat. In the case of CM-based fermented bioproducts, the highest glucosamine yield was obtained after fermentation with 0% of animal fat and 2% of Tween^®^ 40 addition (24.2 mg of glucosamine/g of bioproduct, Figure 2).

### 3.6. Antioxidant Activity

Increased content of total lipids in different forms or structures may lead to their faster degradation. Two types of extracts, namely water and ethanol, were investigated. Obtained results showed that the addition of Tween^®^ 40 led to the improved antioxidant activity of the fermented bioproducts, regardless the type of cereal substrate used (Figure 2). Moreover, despite the concentration of animal fat addition, the antioxidant activity of the obtained fermented bioproducts increased after the fermentation. This was valid mainly in water leachates. In the case of the ethanol extracts and WB-based fermentations the oxidant stability increment was not statistically important, regardless of the concentration of Tween^®^ 40 or animal fat. After CM-based fermentations, increased antioxidant activity was observed in all fermented bioproducts, for both water and ethanol extracts (Figure 2).

### 3.7. Monitoring SFF Bioproducts by FTIR-ATR Spectroscopy

**Figure 3:**
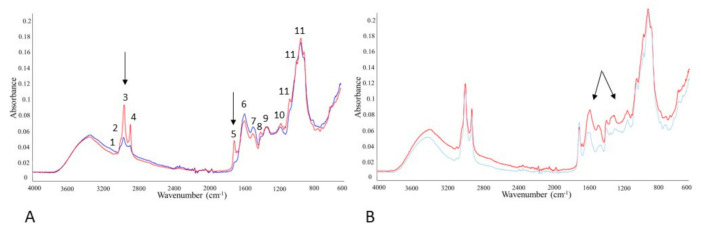
Raw FTIR-ATR spectra of bioproduct obtained by solid-state fermentation of *Umbelopsis isabellina* CCF2412. (**A**) Spectra of fermented wheat bran-based bioproduct with 0% animal fat (blue line) and 30% animal fat addition (red line); (**B**) Spectra of non-fermented (blue line) and fermented (red line) CM-based biorpoduct. Peak numbers correspond to: (1) =C-H stretching in lipids; (2) -C-H (CH_3_) stretching in lipids and hydrocarbons; (3) -C-H (CH_2_) stretching; (4) CH_2_/CH_3_ stretching in lipids and hydrocarbons; (5) C=O ester bond stretching in lipids, esters and polyesters; (6) -C=O stretching, α-Helix Amide I in proteins; (7) N-H bending and C-N stretching, Amide II in proteins; (8 and 9) CH_2_/CH_3_ deformations in lipids; (10) –P=O stretching of phospholipids, polyphosphates and nucleic acids; (11) C-O-C/C-O stretching in polysaccharides; peak assigned according to Kosa et al. [42] and Dzurendova et al. [43]. Black arrows indicate significant changes in lipid-specific region (A) and polysaccharides region (B).

FTIR-ATR spectroscopy was utilized for monitoring biochemical profile of SFF bioproducts. Different IR spectral regions representing information about lipids, proteins, phosphate-containing molecules, and polysaccharides (Figure 3). Clear changes in spectral regions indicate differences in biochemical composition of analyzed SSF bioproducts samples, where the most significant changes were observed for lipid and polysaccharide regions (Figure 3). Subsequent PCA of FTIR-ATR spectra proved distinct variations between analyzed fermented bioproducts in accordance with different composition of samples. High sensibility and accuracy of FTIR-ATR method was mirrored in a clear grouping of samples mainly in order of animal fat concentration. However, the large effect of different animal fat additions was not the only variable affecting biochemical composition of SFF bioproducts that was observed by FTIR-ATR (highlighted by the arrows on the Figure 3A). Formed FBM contains high levels of polysaccharides, that can be observed in the region 1200–900 cm^−1^ [40,41] of obtained FTIR-ATR spectra (highlighted by the arrows on the Figure 3B). Changes in this region correspond with enhanced fungal growth during the SSF process.

## 4. Discussion

The ability of filamentous fungus *Umbelopsis isabellina* to accumulate a high amount of lipid has been previously thoroughly described [16,17,35]. Since this specific lipid contains large levels of n-6 polyunsaturated fatty acids (PUFAs), especially γ-linolenic acid, its microbial overproduction represents a potentially interesting way of their preparation with possible direct application in various industries. The main goal of this study was to find fungal producer alternative to previously described [13] *M. alpina*, which can improve the yield of the produced microbial lipophilic compounds, mainly n-6 fatty acids, during SSF. The addition of animal fat material into the SSF system strongly affected the growth of fungal biomass. With increasing concentration of added animal fat material, the availability of the wheat bran and cornmeal matrixes, which provides basic growth factors to the cultivated *U. isabellina*, deteriorated. A higher concentration of animal fat may also cause changes in crucial bioengineering characteristics of the SSF system (i.e., aeration throughout solid matrix). A similar effect of animal fat addition on FBM growth was observed during our previous study [13] dealing with the possible application of *M. alpina* for biotransformation of animal fat by-product material. Similar to the *U. isabellina* growth characteristics, mainly concentrations of animal fat higher than 20% caused a rapid decrease of FBM formation. These characteristics seemed to be coupled with SSF processes only since the limited addition of various exogenous oils into submerged media is in general described as strongly positive for fungal biomass formation [6,44,45]. Overall, the application of various animal-based fatty substrates for fermentation processes, although mostly submerged type of fermentation, has been reported as very promising. The various strains tested (mostly yeast-like) are defined as a powerful tool for biotransformation of input exogenous FAs, resulting in significant microbial growth and subsequent accumulation of cellular storage lipids [27,28,46,47].

Fungal strain *U. isabellina* is a well-known producer of various FAs [16,17]. This ability was also confirmed during this study. Thanks to the effective desaturase biosynthesis system, *U. isabellina* was able to produce long-chain PUFAs, mainly GLA. Due to that SSF substrates contained two types of carbon source, sugar-based from cereal matrixes and fat-based from animal fat materials, we can assume that both de novo and ex novo production of PUFA run at the same time.

Along with the increasing concentration of animal fat in the SSF system, the percentage of total saturated and monosaturated FAs in the non-fermented animal fat containing SSF substrate was significantly higher in comparison to the control SSF substrates containing only cereal matrixes and 0% of animal fat addition. However, the index of fatty acid unsaturation of obtained fermented bioproducts increased after the SSF process with higher (10, 20 and 30%) addition of animal fat. Since added animal fat by-product material is rich in saturated and monounsaturated fatty acids (Table 1), it indicates efficient biotransformation of waste animal fat material to high-value long-chain PUFA. After the SSF process, elevated levels of GLA in fermented products were observed that resulted in an increased index of fatty acid unsaturation (IU; Table 4) in total fermented products. Similar characteristics of IU changes were described previously during fermentation processes employing other fungi from Mucorales order [10,48]. Different characteristics of IU changes were observed during fermentation using yeast *Yarrowia lipolytica*, which is the most common microorganism used for the possible biotransformation of various fatty substrates [24,49,50]. Generally, a gradual decrease in the IU of the cellular lipid produced was observed in most cases [25,28,51]. This can be caused by either partial inactivation of specific desaturases or direct incorporation of fatty acid-based substrates into intracellular storage lipids. It finally results in the accumulation of saturated fatty acids especially into triacylglycerols that are not further transformed to individual long-chain PUFAs.

During this study, *U. isabellina* was able to accumulate up to 2.6 mg GLA/g of fermented bioproduct in the case of pure wheat bran substrate without animal fat addition. However, after animal fat addition, the yield of GLA increased up to 3.2 mg GLA/g of fermented bioproduct (in the case of 20% animal fat addition). It should be mentioned, that GLA production capacity of *U. isabellina* is even higher. Accumulation up to 11.5 mg GLA/g of dry substrate was observed during optimized conditions described in our previous study [17]. Nevertheless, the GLA yields reached in this study are still fully comparable with production ability of other Zygomycetes strains, which confirms excellent GLA production capacity of *U. isabellina* not only during common submerged fermentation, but also in SSF [16,52,53]. This study proved previously reported excellent ability of various wild-type *U. isabellina* strains for effective single cell oil (SCO) production. In general, conversion yields of input carbon sources to produced lipid in FBM are similar to the maximum ones achieved for genetically engineered SCO-producing strains [54]. When expressing the GLA content related to pure FBM, the positive effect of added animal fat is even more pronounced. The increase in GLA yield was more than 3-fold in this case (from 8.3 mg GLA up to 27.3 mg GLA/g of FBM). However, in the case of CM-based SSF processes, the GLA yield was even higher and reached value up to 6.9 mg GLA/g of fermented bioproduct. 

Various Zygomycetes strains have also been previously described as good producers of carotenoid pigments during SSF [8,16,34]. This ability was one of the key characteristics of why to choose strain *U. isabellina* for possible biotransformation of available animal fat material. The assumption that fermented bioproducts will be simultaneously enriched with both essential PUFAs and carotenoid pigments, mainly β-carotene, is crucial for possible further industrial application, where the production of two or more high-value metabolites is emphasized. After CM-based SSF, the accumulation of β-carotene in the obtained fermented bioproducts reached comparable values to previously published studies [17]. For example, in the case of CM mixtures with Tween^®^ 40 (regardless of the concentration of Tween^®^ 40) and in CM mixtures with moderate animal fat addition (5 and 10%), good accumulation of β-carotene up to more than 30 μg/mg of fermented bioproduct was recorded. With increased animal fat concentration in the SSF system, β-carotene yield in the obtained bioproduct decreased. However, it is necessary to mention, that β-carotene content in fungal biomass was still statistically significant even in the case of 20 and 30% animal fat addition. On the other hand, despite initial assumptions, β-carotene yields reached only trace amounts in the case of WB-based fermentations, regardless animal fat concentration in the SSF system. This is in opposite to our previous findings, where β-carotene yields after WB-based SSF reached 1.5 μg/mg of fermented bioproduct [17]. All tested cereal substrates served as sources of all components necessary for fungal proliferation, such as assimilable carbon, which is indispensable in fungal cell growth and subsequent lipid storage [55,56]. Chemical composition (i.e. the percentage of polysaccharides, β-glucans or cellulose [57]) of these cereal sources might vary year-to-year, which is given by natural changes in weather or microclimate of cultivation of these agricultural products. However, these small changes in chemical composition possibly strongly affect performed SSF processes. Further investigation to understand carotenoid biosynthesis pathway blockage in WB-based SSF is the next goal of our research.

In addition to β-carotene production, enhanced glucosamine accumulation in formed FBM was observed in the case of both CM- and WB-based fermented bioproducts obtained from SSF of control CM and WB substrates without animal fat supplementation. This predetermines the application of obtained fermented bioproducts in further industrial processing, not only feed or food but also in pharmaceutical or cosmetic fields. Microbial glucosamine production is still not in favor to traditional production by hydrolysis of crustacean shells [58,59], improved fermentation production offers the possibility to circumvent problems associated with such bioproduction. Nevertheless, it should be mentioned that especially higher additions of animal fat material (20% and 30%) into SSF substrate mixtures led to the decrease in fungal growth, which caused a worsening of glucosamine yields.

One of the problems that limit the range of a wider direct industrial application of different fermented products enriched with less stable compounds, such as long-chain PUFAs, can be the possible oxidation of such products. Antioxidant activity characterizes in general the resistance or protection of fats and oils to oxidation. The ability of fermentation processes to improve antioxidant activity is well described [60,61,62]. It is primarily due to an increased amount of flavonoids and various phenolic compounds that is the result of a microbial hydrolysis of cereal substrates. It finally leads to the synthesis of various antioxidant compounds [60,63], such as carotenoid pigments in our case. After Tween^®^ 40 addition to the SSF system, regardless its concentration, increased antioxidant activity was observed. Tween^®^ 40 acts as a detergent. Thus, in addition to the aforementioned ability to reduce the surface tension between water and fat phases, Tween^®^ 40 increases the ability of substances to penetrate aqueous solutions. FBM growth during SSF is accompanied by several significant changes in the composition of cereal substrate, such as higher content of extracellular proteins (that are usually present in water extracts and which can excel the radical quenching well), carotenoids (ethanol extracts), etc. These compounds are known as very good antioxidants [64]. Thus, the increased content of such compounds is responsible for improved antioxidant activity of obtained fermented bioproducts. This is also proved by antioxidant activity measured in WB-based extracts since the results showed that *U. isabellina* does not have as good growth rate compared to CM-based SSF. Moreover, there was no β-carotene formation observed, which proved these findings. However, increased antioxidant activity in ethanol extracts was minimal in the case of CM-based fermented bioproducts. In the case of WB-based fermented bioproducts, increased antioxidant activity was not observed at all. This is because emerging proteins do not have the ability to penetrate the ethanol phase. A further increase in antioxidant activity can be achieved by an increased content of β-carotene, which is generally considered to be good radical scavengers. Initiation of carotenoids biosynthesis, and their subsequent overproduction during SSF is the next future goal of our research.

## 5. Conclusions

The presented study is the first one dealing with the effective utilization of AF by-products into functional high-valued fermented bioproducts by SSF of *Umbelopsis isabellina* CCF2412. AF used as an addition to the cereal matrixes was to some extent successfully transform into other fat-based compounds. Obtained fermented bioproducts were enriched not only with high-valued n-6 γ-linolenic acid but also with glucosamine and β-carotene. Further, it was mainly β-carotene that caused improved oxidative stability of obtained fermented bioproducts. Increased antioxidant activity is one of the crucial characteristics that predetermines the good applicability of fermented products with desired quality in industrial applications. Further optimization of such fermentation process can easily lead to increased yields of mentioned specific fungal metabolites. Successful application of FTIR-ATR spectroscopy for rapid analysis of *U. isabellina* fermented bioproducts has also been proved, which can possibly extend potential of the application of this rapid method for analysis of fermented products.

## Figures and Tables

**Table 1:** Fatty acid (FA) composition and percentage of individual lipid components of animal fat material used for the solid-state fermentation by *Umbelopsis isabellina* CCF2412.

FA	[%]	Lipid Structure	[%]
C14:0	2.23		
C16:0	25.66	Polar lipids	0.14
C16:1 n-7	2.18	Monoacylglycerols	0.28
C18:0	21.33	Diacylglycerols	2.08
C18:1 n-9	38.34	Sterol structures	9.38
C18:1 n-7	1.94	Free fatty acids	7.20
C18:2 n-6	5.47	Triacylglycerols	66.54
C18:3 n-3	0.81	Esterified sterols	11.23
C20:0	0.23	Other lipid structures	3.15
Other FAs	1.81		

**Table 2:** Amount of added Tween^®^ 40 and animal fat to cereal matrixes used for the solid-state fermentation by *Umbelopsis isabellina.*

Cereal Matrix	Tween^®^ 40 [%(w/w)]	Animal Fat [%(w/w)]
wheat bran/cornmeal (20 g)	0	0
	0.5	0
	1	0
	2	0
	3	0
	0.5	5
	1	10
	2	20
	3	30

**Table 3:** The percentage of cereal matrix utilization and SSF substrate humidity, pH and water activity (a_w_) values, and fungal biomass (FBM) content in fermented bioproducts obtained by SSF of animal fat (AF) material by *Umbelopsis isabellina* CCF2412.

	Cereal Matrix Utilization [%]	Fermented Bioproduct Humidity [%]	a_w_ [Non-Fermented Substrate]	a_w_ [Fermented Bioproduct]	pH [Fermented Bioproduct]	FBM [mg/g BP]
Cornmeal (CM)	22.0 ± 1.5	62.0 ± 1.9	0.959 ± 0.014	0.957 ± 0.004	5.1 ± 0.1	242.7 ± 12.5
CM + 0.5% Tween 40	21.2 ± 0.8	60.5 ± 0.3	0.958 ± 0.008	0.959 ± 0.007	4.9 ± 0.0	247.2 ± 19.8
CM + 1% Tween 40	21.9 ± 1.4	62.6 ± 1.2	0.957 ± 0.020	0.962 ± 0.005	4.5 ± 0.3	250.6 ± 13.6
CM + 2% Tween 40	24.7 ± 1.6	60.0 ± 1.9	0.958 ± 0.009	0.964 ± 0.011	4.2 ± 0.2	248.3 ± 7.9
CM + 3% Tween 40	28.5 ± 1.6	62.3 ± 1.3	0.960 ± 0.010	0.965 ± 0.001	4.1 ± 0.1	231.8 ± 15.7
CM + 0.5% Tween 40 + 5% AF	25.4 ± 1.9	60.6 ± 1.5	0.958 ± 0.017	0.964 ± 0.004	4.6 ± 0.1	206.2 ± 19.4
CM + 1% Tween 40 + 10% AF	23.8 ± 1.0	54.8 ± 0.6	0.957 ± 0.007	0.966 ± 0.014	4.2 ± 0.1	151.4 ± 16.0
CM + 2% Tween 40 + 20% AF	12.9 ± 1.1	47.7 ± 1.8	0.951 ± 0.005	0.967 ± 0.011	4.3 ± 0.1	113.4 ± 9.7
CM + 3% Tween 40 + 30% AF	6.6 ± 0.1	38.5 ± 1.6	0.943 ± 0.005	0.964 ± 0.014	3.9 ± 0.1	93.0 ± 1.0
Wheat Bran (WB)	17.3 ± 0.3	59.7 ± 0.4	0.933 ± 0.027	0.938 ± 0.025	4.6 ± 0.5	229.9 ± 10.4
WB + 0.5% Tween 40	17.4 ± 0.3	59.4 ± 1.0	0.933 ± 0.015	0.940 ± 0.007	5.1 ± 0.6	255.1 ± 8.9
WB + 1% Tween 40	17.4 ± 0.3	59.3 ± 0.4	0.932 ± 0.037	0.941 ± 0.015	5.3 ± 0.3	253.3 ± 7.4
WB + 2% Tween 40	19.5 ± 0.9	59.1 ± 0.6	0.934 ± 0.016	0.941 ± 0.008	5.3 ± 0.2	257.2 ± 2.9
WB + 3% Tween 40	21.8 ± 0.8	59.0 ± 0.1	0.933 ± 0.004	0.943 ± 0.014	5.4 ± 0.3	266.9 ± 2.4
WB + 0.5% Tween 40 + 5% AF	15.8 ± 1.0	56.4 ± 1.3	0.939 ± 0.025	0.939 ± 0.005	5.1 ± 0.1	222.1 ± 10.0
WB + 1% Tween 40 + 10% AF	13.8 ± 0.6	52.4 ± 1.0	0.937 ± 0.017	0.934 ± 0.018	5.0 ± 0.1	171.9 ± 18.2
WB + 2% Tween 40 + 20% AF	10.3 ± 0.3	46.4 ± 0.6	0.928 ± 0.015	0.925 ± 0.011	4.6 ± 0.4	143.3 ± 10.0
WB + 3% Tween 40 + 30% AF	8.9 ± 0.6	39.7 ± 0.2	0.924 ± 0.009	0.921 ± 0.015	4.5 ± 0.2	113.3 ± 1.7

**Table 4:** Percentual main fatty acids composition, total fatty acid content (TFA), and index of unsaturation (IU) of non-fermented (nf) and fermented (f) SSF substrates (cornmeal—CM, wheat bran—WB) with the addition of different amounts of Tween^®^ 40 (T) and animal fat material (AF). The results are an average of three independent biological replicates with α < 5%.

	IU	TFA	Fatty Acids (%)						
		(%/BP)	C16:0	C16:1, n-7	C18:0	C18:1, n-9	C18:2, n-6	C18:3, n-6	Other FAs
**Cornmeal**									
CM nf	1.19	3.5	13.2	0.0	2.4	27.1	55.2	0.0	2.1
CM f	0.69	9.9	18.6	1.6	3.3	44.0	22.8	5.3	4.4
+ 0.5% Tween 40 nf	1.15	3.5	15.3	0.0	2.5	26.3	52.8	0.0	3.1
+ 0.5% Tween 40 f	0.68	11.5	19.5	1.6	3.3	43.8	22.3	5.2	4.3
+ 1% Tween 40 nf	1.15	4.2	15.8	0.0	2.4	26.2	51.9	0.0	3.7
+ 1% Tween 40 f	0.74	11.4	19.7	1.5	3.4	42.9	22.7	5.6	4.2
+ 2% Tween 40 nf	1.09	4.1	21.5	0.0	2.6	23.9	48.2	0.0	3.7
+ 2% Tween 40 f	0.71	13.5	21.1	1.5	3.5	43.3	21.3	5.1	4.1
+ 3% Tween 40 nf	1.13	4.5	24.3	0.0	2.6	21.9	47.9	0.0	3.3
+ 3% Tween 40 f	0.76	13.9	22.4	1.5	3.6	41.3	21.8	5.4	4.0
+ 0.5% T + 5%AF nf	0.78	6.8	20.7	1.2	10.7	32.6	30.3	0.0	4.4
+ 0.5% T + 5%AF f	0.71	15.2	20.1	1.8	7.1	43.3	18.3	4.7	4.8
+ 1% T + 10%AF nf	0.46	10.7	24.3	1.6	14.8	34.5	19.5	0.0	5.3
+ 1% T + 10%AF f	0.49	17.6	21.2	1.9	8.5	43.7	16.1	3.6	5.0
+ 2% T + 20%AF nf	0.40	15.4	25.1	1.8	15.9	35.6	16.1	0.0	5.6
+ 2% T + 20%AF f	0.40	19.9	22.8	2.0	12.1	42.2	13.7	1.9	5.4
+ 3% T + 30%AF nf	0.31	21.7	27.2	2.0	17.8	35.5	12.0	0.0	5.5
+ 3% T + 30%AF f	0.35	22.4	24.4	2.1	14.9	39.7	12.5	0.9	5.5
**Wheat Bran**									
WB nf	1.46	3.6	17.1	0.0	1.0	17.3	56.1	0.0	8.5
WB f	1.41	3.4	14.4	0.5	2.1	29.0	40.3	7.5	6.3
+ 0.5% Tween 40 nf	1.42	3.6	20.7	0.0	1.1	16.6	54.7	0.0	6.8
+ 0.5% Tween 40 f	1.40	2.8	14.5	0.5	2.3	29.1	38.8	8.3	6.5
+ 1% Tween 40 nf	1.39	3.8	21.8	0.2	1.2	16.7	53.4	0.0	6.8
+ 1% Tween 40 f	1.40	2.8	14.6	0.5	2.3	29.6	37.8	8.5	6.8
+ 2% Tween 40 nf	1.31	3.8	26.7	0.0	1.5	15.8	50.1	0.0	6.0
+ 2% Tween 40 f	1.39	2.6	15.4	0.5	2.4	28.7	38.1	8.3	6.6
+ 3% Tween 40 nf	1.17	4.6	33.6	0.1	1.8	13.7	44.9	0.0	5.9
+ 3% Tween 40 f	1.35	3.1	17.2	0.6	2.5	28.5	37.6	7.3	6.3
+ 0.5% T + 5%AF nf	1.08	6.6	22.1	1.1	9.5	26.5	34.6	0.0	6.3
+ 0.5% T + 5%AF f	1.21	4.6	17.2	1.1	5.0	36.1	28.1	6.3	6.1
+ 1% T + 10%AF nf	0.99	9.9	23.7	1.2	10.6	27.2	30.8	0.0	6.6
+ 1% T + 10%AF f	1.02	6.8	19.1	1.5	8.9	37.9	22.3	4.3	6.1
+ 2% T + 20%AF nf	0.83	14.4	25.8	1.7	12.8	31.1	22.2	0.0	6.4
+ 2% T + 20%AF f	0.86	11.7	22.3	1.7	11.4	40.6	15.4	2.7	5.9
+ 3% T + 30%AF nf	0.76	23.4	26.1	1.6	16.0	33.3	17.1	0.0	6.0
+ 3% T + 30%AF f	0.83	20.0	23.5	1.7	11.2	40.6	15.8	1.6	5.6

**Table 5:** TLC results describing the percentual representation of individual lipid classes in non-fermented substrate mixtures (nf) and fermented bioproducts (f) prepared by SSF of *Umbelopsis isabellina* CCF2412 with the addition of different amounts of Tween^®^ 40 (T) and animal fat (AF). The results are an average of three independent biological replicates with α < 5%.

	Lipid Class (%)							
	Polar Lipids	1,2-Diacylglycerols	Sterols	Free Fatty Acids	Coenzyme Q	Triacylglycerols	Esterified Sterols	Others
**Cornmeal**								
CM nf	6.0	5.6	5.1	3.9	2.1	66.2	9.2	1.9
CM f	4.7	8.8	11.8	12.1	0.8	50.0	11.1	0.7
+ 0.5% Tween 40 nf	4.7	3.4	4.0	4.2	2.0	71.6	8.0	2.0
+ 0.5% Tween 40 f	4.3	7.1	9.7	11.5	1.2	53.0	12.1	1.1
+ 1% Tween 40 nf	4.6	2.8	3.7	4.1	1.2	72.9	8.5	2.4
+ 1% Tween 40 f	4.3	5.6	7.6	13.8	1.1	56.4	10.5	0.7
+ 2% Tween 40 nf	3.8	3.1	5.2	5.3	1.8	71.2	7.7	1.9
+ 2% Tween 40 f	5.3	6.3	10.9	11.9	1.2	51.5	11.8	1.1
+ 3% Tween 40 nf	3.7	2.7	4.1	7.2	2.6	69.9	8.7	1.2
+ 3% Tween 40 f	5.5	6.2	7.7	14.2	0.7	53.7	11.1	0.9
+ 0.5% T + 5%AF nf	4.1	2.8	2.5	4.8	1.1	74.2	5.7	5.0
+ 0.5% T + 5%AF f	4.1	7.0	8.2	13.2	0.8	55.7	8.5	2.5
+ 1% T + 10%AFnf	4.2	1.0	2.8	6.8	0.9	69.8	4.4	10.2
+ 1% T + 10%AF f	3.8	5.3	6.9	13.5	0.7	59.9	7.7	2.3
+ 2% T + 20%AF nf	4.1	5.1	2.7	6.6	0.3	60.1	4.1	16.2
+ 2% T + 20%AF f	3.9	5.4	9.3	12.5	0.3	56.8	5.1	6.9
+ 3% T + 30%AF nf	6.1	2.4	2.3	7.5	0.3	63.7	3.7	14.1
+ 3% T + 30%AF f	3.4	5.2	3.9	12.0	0.0	66.9	3.5	5.1
**Wheat Bran**								
WB nf	8.8	9.1	3.2	6.6	0.6	45.5	13.3	13.1
WB f	8.4	7.4	10.4	16.6	1.9	24.2	19.5	11.6
+ 0.5% Tween 40 nf	9.6	10.2	3.3	8.8	0.2	45.9	7.9	14.1
+ 0.5% Tween 40 f	11.0	7.8	11.4	14.5	1.8	27.0	13.1	13.5
+ 1% Tween 40 nf	8.8	8.4	3.5	8.1	0.3	45.9	13.2	11.9
+ 1% Tween 40 f	12.2	7.1	11.9	14.0	1.8	27.7	17.8	12.5
+ 2% Tween 40 nf	9.8	14.2	5.5	6.1	0.0	47.3	8.2	8.9
+ 2% Tween 40 f	11.5	7.4	15.1	13.5	1.6	22.9	16.7	13.2
+ 3% Tween 40 nf	10.1	14.2	5.9	6.0	0.2	43.6	11.6	8.5
+ 3% Tween 40 f	10.9	7.4	15.2	10.3	1.6	30.7	13.0	10.9
+ 0.5% T + 5%AF nf	7.3	10.8	9.4	7.6	0.0	50.3	7.7	7.0
+ 0.5% T + 5%AF f	8.7	7.2	10.9	15.9	1.1	31.7	16.1	8.5
+ 1% T + 10%AF nf	8.7	10.1	9.5	7.5	0.0	52.0	6.2	6.1
+ 1% T + 10%AF f	8.5	7.1	11.6	16.8	0.7	38.3	11.8	5.2
+ 2% T + 20%AF nf	6.0	7.2	10.0	9.1	0.0	61.9	3.4	2.5
+ 2% T + 20%AF f	7.4	6.3	10.5	17.6	0.3	47.7	7.0	3.3
+ 3% T + 30%AF nf	5.8	6.9	8.2	9.2	0.0	61.3	5.3	3.3
+ 3% T + 30%AF f	7.5	6.3	10.8	15.9	0.0	49.7	6.9	3.0

## Data Availability

The data presented in this study are available on request from the corresponding author.
